# Microscopical Researches of Dr. Th. Schwann into the Structure of the Teeth

**Published:** 1851-04

**Authors:** 


### ARTICLE XVIII.

### *Microscopical Researches of* DR. TH. SCHWANN *into the Structure of the Teeth.*

2. *The Teeth.*—The teeth were formerly classed with the bones, but have of late been treated of as non-vascular structures, under the head of horny tissues. Since Miescher's discovery, however, that the vessels of bone also traverse only the medullary canaliculi, since Müller observed that the teeth, like the bones, afford gelatine by boiling, and Retzius discovered osseous corpuscles in the ivory, it seems more correct to class the teeth with the bones again, and the more so, as we now know that the presence or absence of vessels proves no *essential* difference in the growth. The coalescence of the cell-walls which appears to take place in the ivory of the teeth forms an additional reason for our classing them with bone. The teeth, as is well known, consist of ivory, osseous substance, and enamel.

### *The Enamel.*

The enamel consists, according to Purkinje, of square, or, according to Retzius, of hexagonal closely-aggregated prisms, which stand nearly perpendicular upon the surface of the ivory, and pass outwards in a slightly curved direction. It is at first soft, and if some of it be scratched off in that state, we obtain, what Müller has described as needle-shaped bodies pointed at both extremities. According to Purkinje, Raschkow and Retzius, some organic substance remains after the young enamel has been treated with hydrochloric acid, whilst Berzelius asserts that the enamel of mature teeth does not contain two per cent. of organic matter. For further details, I refer the reader to the excellent works of Purkinje, Raschkow and Fränkel, and those of Retzius, J. Müller and v. Linderer.

If an immature tooth of a child or mammal, (the pig, for instance,) be removed from its capsule, and placed in dilute hydrochloric acid, the organic substance of the enamel which remains after the solution of the earthy matter, may be separated from the ivory entire. It has exactly the form and size of the enamel previous to the action of the acid. It is very soft, and breaks readily in the direction of the fibres of the enamel. Examined with a high magnifying power and subdued light, it is found to be composed, like the enamel itself, of closely-aggregated prisms, which may be insulated from one another, so that each one forms an independent structure. This organic substance, therefore, cannot be, as Raschkow and Retzius considered, a mere deposit from the moisture with which the enamel-fibres are at first surrounded, and thus a sort of cast of the enamel-fibres; but either the fibres must result from an ossification of these prisms, or the prisms must be hollow, and the inorganic substance deposited within them. When the enamel of the pig's tooth is examined with a subdued light, the contour of these organic prisms is found to be so dark, in comparison with their interior, that it can scarcely be regarded as the mere shaded outline of a solid prism, but suggests the idea of a cavity surrounded by a thin membrane. This distinction is, however, much less striking in human teeth, so that the question as to which of the two views is correct, must remain undecided.

What, then, is the process of formation of these enamel-prisms? According to Purkinje and Raschkow, the crown of the growing tooth is surrounded externally by a peculiar membrane, the enamel-membrane, the inner surface of which is composed of short hexagonal fibres, which stand perpendicularly upon the membrane, and are directed towards the enamel, so that each fibre of the enamel-membrane corresponds to an enamel-fibre. On examining a portion of this membrane, particularly that part which lies nearest the root of the tooth, we readily recognize in it the characteristic nuclei, some of them being furnished with nucleoli. They lie in a minutely granulous substance. This granulous aspect, however, is seen to be produced, in many situations, by granulated cells which contain the nuclei. Each nucleus is surrounded by a circular areola of small granules, and seems to lie in a minutely granulated globule, which we know to be the rudimentary form of most elementary cells. Some of these cells are prolonged into very delicate fibres; they appear to be young cells of areolar tissue; most of them, however, are round. The fibres or prisms of the membrane, which have a direction from its inner surface towards the enamel-fibres, have assumed a hexagonal form, which Raschkow attributes to their close contact. They very closely resemble the columnar epithelium upon mucous membranes, only that they are prismatic in their entire length, that is, so far as they project out from the membrane to which they are attached. I am inclined, therefore, to regard them as merely elongated cells. In the recent state, they also contain a very distinct nucleus, which encloses its nucleolus. In the upper part of the enamel-membrane they lie quite close together; but in the portion nearest to the root of the tooth, they diminish in number, and stand insulated, so that at this part the structure of the membrane beneath them may also be recognized, and I suppose the round cells before mentioned to be the earlier condition of these prismatic cells. What, then, is the relation which these prismatic cells of the enamel-membrane bear to the prisms of the enamel? Purkinje and Raschkow regarded each fibre of the enamel-membrane as an excretory organ, a little gland which secreted the enamel-fibre corresponding to it. With our altered views of the growth of unorganized* tissues, however, this explanation, previously so plausible, loses much of its probability. Various other explanations might be offered in place of it, but I have not made sufficient observations to enable me to decide upon the correct one. Firstly, one might suppose the organic basis of the enamel-prisms to be cells, which are formed, and continue to grow independently upon the dental substance, having no other connection with the prisms of the enamel-membrane than that the latter furnishes their cytoblastema. This explanation, however, would compel us to regard the remarkable accordance which exists between the prisms of the enamel-membrane and those of the enamel as an accidental circumstance. But we should be obliged to adopt such a view, if it could be proved that another peculiar substance intervened between the enamel-membrane and the enamel, and I have several times observed such an one on the molar teeth of swine. It is very soft and full of vesicles, having the appearance of a slag. I think Purkinje mentions it also, but I cannot find the precise passage at this moment. It lays between the enamel-membrane and the

* [The author appears to use this word as synonymous for "non-vascular."—Trans.]

tooth, but I am not certain whether it was also present at those points where the formation of the enamel had already commenced, and whether, therefore, it actually interrupted the continuity of the enamel-membrane with the formed enamel. We might suppose, as a second explanation, that the enamel-prisms are uninterrupted continuations of the prisms of the enamel-membrane, which become filled towards one end with calcareous salts. This is a very improbable explanation, and the connection between the two structures is of too loose a nature to warrant its adoption. A third, and as I am at present disposed to think the most probable, explanation, is that the prismatic cells of the enamel-membrane separate from it, and coalesce with the enamel already formed, while at the same time their cavities either become filled with calcareous salts, or they become ossified throughout their entire thickness, their cavity being previously filled with an organic substance. This explanation makes the formation of the enamel accord with the growth of the other unorganized tissues treated of in the previous class. If we suppose, for example, that the little cylinders, (columnar epithelium) of the mucous membranes, (which, according to Henle, are constantly being thrown off,) could become ossified at the moment when they separated from the surface of the mucous membrane, we should obtain a covering to the membrane, consisting of little calcareous cylinders, each of which, however, would still have its organic basis like the enamel-fibres. Beneath this covering would be other cylinders not as yet ossified, which, when they in like manner became calcified, would add to its thickness, whilst new cylinders grew forth from the mucous membrane. The quantity of the organic basis is extremely small in the teeth of adults which have been exposed for a considerable time to the action of the saliva, a circumstance which I suppose to be referable to its undergoing a chemical solution in that fluid.

### *The Ivory.*

This is known to consist of a structureless substance, traversed by a great many minute canals. These canals, (tubes,)

have for the most part a radiate course from the cavity of the tooth towards its external surface, and, according to Retzius, often give off branches as they proceed. Their peripheral terminations are extremely minute; they are thicker towards the dental cavity, and, when the pulp is removed, open freely into it. Müller observed that the tubes projected beyond the intermediate substance from the fractured surface of thinly-ground laminæ, and of lamellæ which had been macerated in hydrochloric acid, and were surrounded, therefore, by a special membrane; Retzius also remarked the same upon a transverse section. Purkinje and Müller noticed that when teeth are placed in ink, the fluid penetrates into the tubes; they must therefore be hollow. If any of them contain calcareous matter, it must be only the most minute ones. According to Retzius, many teeth present corpuscles which resemble those of bone, and like them send forth minute radiating canaliculi.

What relation, then, does the ivory bear to the cells? I must at once avow that I cannot give a positive reply to this question, and that I only communicate the following imperfect investigation for the sake of presenting a connected view of my subject. The formation of the dental substance is described by Purkinje and Raschkow as follows: "Primordio substantia dentalis e fibris multifariam curvatis convexis lateribus sese contingentibus ibique inter se concrescentibus composita apparet. . . . . In ipso apice istæ fibræ æqualiter quamcunque regionem versus se diffundunt, attamen parietes laterales versus directio longitudinalis prævalet, dum fibræ sinuosis flexibus æqualique modo se invicem contingentes ibique ubi concavæ apparent lacunas inter se relinquentes, ab apice coronali radicem versus ubicunque procedunt. Non nisi extremi earum fines tunc molles sunt ceteræ autem partes brevissimo tempore indorescunt. . . . . Substantiæ dentalis formationis secundum crassitudinem processus pari modo ac primo ejus ortu cogitandus est. Postquam . . . . fibrarum dentalium stratum depositum est, idem processus continuo ab externa regione internam versus progreditur, germinis dentalis parenchymate materiam suppeditante. . . . . Convexæ

30*

fibrarum dentalium flexuræ, quæ juxta latitudinis dimensionem crescunt, dum ab externa regione internam versus procedunt, sibi invicem appositæ continuos canaliculos effingunt, qui ad substantiæ dentalis peripheriam exorsi multis parvis anfractibus ad pulpam dentalem cavumque ipsius tendunt, ibique aperti finiuntur, novis ibi, quamdiu substantiæ dentalis formatio durat, fibris dentalibus aggregandis inservientes."—(Raschkow, *Meletemata circa Mammalium Dentium Evolutionem.* Vratislav., 1835, p. 6.)

I must admit that I do not clearly understand some of this description, but if I rightly comprehend it, the dental substance originates from fibres which are formed in strata around the pulp, (the latter supplying the material for the purpose;) that these fibres then coalesce, leaving, however, spaces between them, which are the dental tubes. Since, according to Müller, the tubes are furnished with special walls, we can no longer regard them as mere spaces between the fibres. His observation, however, does not affect the explanation of the formation of the firm substance.

If a tooth be removed from its capsule, and macerated for some days in slightly diluted hydrochloric acid, the dental substance, which on the first withdrawal of the calcareous salts possessed a cartilaginous consistence, becomes so very soft that it can only be removed from the acid in very small portions with the forceps. This pappy mass is found, on examination, to consist of fibres, which may here and there be insulated. These fibres are too thick to be the walls of the tubes; they form the entire substance. Nor can they well be an artificial product, the result of the acid penetrating into the tubes, and dissolving, in the first instance, the substance in immediate contact with them, so that the intercellular substance remained undissolved in the form of a fibre; they are too regular and smooth for that. It appears rather that the dental substance is composed of these fibres, which have become blended together, that they are therefore identical with those fibres, by the coalescence of which, according to Purkinje and Raschkow, the dental cartilage is formed, and that this coalescence is not so complete, but that it may be artificially dissolved. The fibres have the same course as the tubes in human teeth, but I could no longer perceive the tubes between them in this preparation; I could, however, recognize the fibres everywhere, save in the most external layers, which lay immediately under the enamel, in which situation the mass was more completely broken down by the acid, and traversed by more minute fibres of a different kind, having the most confused and varied directions, and which I suppose to have been the remains of the dental tubes.

We must therefore regard the dental substance as composed of fibres blended together, between which run tubes provided with special walls. The fibres and tubes are nearly perpendicular to the dental cavity in human teeth. What connection now is there between the fibres, or the tubes, and the cells? I should incline to the old opinion, that the dental substance is the ossified pulp. According to Purkinje and Raschkow, the pulp, in the first instance, consists of globules, of nearly uniform appearance, but has neither vessels nor nerves. At a subsequent period, vessels appear in it, and afterwards nerves. Upon the surface of the pulp, the globules are more regularly arranged, and more extended in the longitudinal direction, and are directed towards the outside perpendicularly, or at a slightly acute angle. These elongated globules are clearly cylindrical cells. In recent teeth, the characteristic nucleus with its nucleoli may be distinctly seen in them, and they very closely resemble the prisms of the enamel-membrane. The interior of the pulp also consists of round nucleated cells, between which the vessels and nerves pass. When the pulp of a young tooth is detached from its cavity, and the dental substance is examined, (without further preparation, or after the earthy matter has been withdrawn,) a stratum of the cylindrical cells of the pulp will be found to remain attached to its internal surface, at least to the lower part of it, where the newly-formed dental substance is yet thin and soft. These cells are of about the same size, and have the same course as the solid fibres of the dental substance; and since, on the one hand, they clearly belong to the pulp, which follows from their accordance with the cylindrical cells that remain attached to the rest of its surface, and as, on the other hand, they are still more firmly connected with the dental substance than with the pulp, and remain affixed to the former, I suppose a transition to take place at that part, and the cylindrical cells of the pulp to be merely the earlier stage of the dental fibres, i. e. that the cells become filled with organic substance, solid and ossified. In some instances, these little cylinders are not found upon the dental substance, but a quantity of cell-nuclei seen in their place; these are very pale, and so intimately united with the dental substance, that they readily escape notice; when, however, attention is once attracted to them, it is impossible to mistake them, and they lie side by side, with extremely small interspaces. The facility with which the two structures may be separated, has been adduced as an argument against the opinion that the dental substance is the ossified pulp, and I fully acknowledge the weight of the objection. But the following circumstances deprive it of at least some of its importance. Firstly, some portion of the pulp actually remains attached to the dental substance; again, in ribs which are half ossified, the cartilage may easily be separated from the ossified portion; and lastly, the separation must be effected with more facility in the tooth, in consequence of the greater difference in consistence between the dental substance and the pulp. There are therefore, at least, reasons enough to warrant our entering more particularly into the details of this opinion. The pulp accords with all the other tissues of the fetus, therefore with cartilage, in being composed of cells: the difference between its consistence and that of the cartilage of mammalia, depends on this, that the quantity of cytoblastema, (to which the latter owes its hardness,) is very small, for the cylindrical cells of the pulp lie quite close together, at least such is the case on its surface. In this respect, the pulp bears a closer analogy to certain cartilages of animals lower in the scale, in which there is also only a small quantity of cytoblastema present, and the consistence of the cartilage is principally occasioned by thickening of the cell-walls. As I have not actually observed the transition, I do not know whether the filling up of the cavities also takes place by thickening of the cell-walls, in this supposed conversion of the cells of the pulp into the dental fibres. If such be really the case, the cavities of the cells are in general so completely obliterated by it, that no cartilage-corpuscles remain. From the observations of Retzius, however, it might be supposed that some of the cells retain their cavities, and even become transformed into stellated cells; for he saw true osseous corpuscles in the dental substance. When the uppermost stratum of the pulp, consisting of cylindrical cells, has become converted into dental substance by ossification, the round cells lying immediately next beneath it in the parenchyma of the pulp, must first commence their transformation into cylindrical cells, the vessels of the stratum must become obliterated, and then this stratum ossified, and so on.

What, then, are the dental tubes? Retzius compares them to the calcigerous canaliculi of bone which issue from the osseous corpuscles, and I was myself at first of that opinion; for I regarded them as prolongations of cells, the bodies of which lay in the pulp. For, when the pulp is drawn out from the cavity of a pig's tooth, and its margins examined, it will be seen that each of the cylindrical cells of the surface of the pulp becomes elongated into a short minute fibre towards the dental substance, and that these fibres are about as numerous as the tubes projecting upon the surface of the pulp. I thought, formerly, that they became elongated to form the dental tubes, and that the intertubular substance was merely intercellular substance between these prolongations. But I was compelled to relinquish that idea, in consequence of there being no such appearance in the human tooth, and because the explanation led to difficulties with respect to the teeth of the pike, in which, according to Retzius, an immediate transition of the dental into the osseous substance takes place. If one of the largest teeth in the lower jaw of the pike be sawn off, deprived of its earthy matter by means of hydrochloric acid, and then divided into thin longitudinal sections, the dental substance will be seen to form a hollow cone, which is filled with osseous substance. The dental substance is transparent, and consists of fibres which have a direction from the point towards the base of the cone. Canals traverse the osseous substance, resembling the Haversian medullary canals of ordinary bone, only they are not so regular. The dental tubes, then, are connected with these canals of the proper osseous substance, and may be distinctly seen issuing from them in a funnel-shaped form. The canaliculi soon ramify in the dental substance, and, as they run across the thickness of the dental cone, interlace with the dental fibres. According to this view, the dental tubes would correspond to the medullary or Haversian canaliculi of bone, and not to the calcigerous canaliculi proceeding from the osseous corpuscles. It appears impossible, however, to be assured of the right explanation of all the structural relations of the dental substance, until its development is examined in teeth differing widely from each other in construction.

### *Osseous Substance of the Teeth.*

This requires no particular explanation, as it entirely accords with the ordinary osseous substance.

Having examined in detail the tissues comprehended in this class, and compared them one with another, we have yet to consider the entire class in relation to those which have been previously discussed, and to observe how much our knowledge of the transformations which the cells are capable of undergoing, has been advanced by the study of it.

It is easy to see which elements of the tissues of this and the preceding class correspond. There the whole tissue consisted of cells, closely crowded together, and the intercellular substance was almost *nil.* Here we find the like arrangement only in the lowest stage of development of the most simple cartilages. In such as are more highly developed, those of all the mammalia for example, the cells lie surrounded by a larger quantity of intercellular substance, which forms the proper cartilaginous substance; but the cell-walls contribute only very slightly, or not at all, to its formation. The proper firm substance of these higher cartilages, therefore, has its analogy in the former class, only in the minimum of cytoblastema by which the cells are connected, while, on the other hand, it corresponds with that which, in the first class, was the fluid, wherein the isolated cells were formed. The cartilage-cells in this class, however, correspond precisely to the epithelium-cells, the feather-cells, &c., &c., in the preceding one, and the blood-corpuscles, mucus-corpuscles, &c., in the first class.

We have not found any new changes in the form of the cells in this class. Most of them were angular, somewhat approaching the circular form; and stellated cell, so far at least as we may be permitted to regard the osseous corpuscles as such, were also frequently met with. Some cells, which were remarkably elongated, were observed near to the surface of several cartilages, in which situation they are known as greatly elongated cartilage-corpuscles; still, however, this appearance is never presented by the cells of this class in so remarkable a degree as it is by those of the crystalline lens in the previous one. The fibro-cartilages, on the other hand, form the immediate transition from this to the following class, for in them a bundle of fibres seems to be formed out of each cartilage-corpuscle, a process which we shall consider more minutely when treating of cellular (areolar) tissue in the next class.

We have observed the formation of cells around the previously-existing nucleus, and their progressive growth, going on in this class in a similar manner to that exhibited in the preceding, and the true cartilage-cells were also seen to form around a cytoblast which lay external to the cells already developed. On the other hand, a formation of cells also takes place within the true cartilage-cells, but it is probable that they have a different signification from those within which they are generated. A deviation from the previous class seemed to occur with respect to the spot at which the young cells are formed, in relation to the entire tissue. In the former class, so far as we could perceive, they were formed at that part only where the tissue was in immediate contact with the organized substance. The formation of the new cells in cartilage, it is true, did not take place throughout the entire thickness of the tissue, but, (so long at least as the cartilage itself is not furnished with vessels,) only near the surface, and therefore, at the spot where it was in contact with the organized substance; still, however, it not only took place at that point of contact, but went on also between the cells most recently formed, as if cartilage had a greater capacity of imbibition, so that the cytoblastema penetrating from the blood-vessels into the parenchyma arrived at the deeper-seated portions of the tissue more speedily; and, therefore, retained its fresh plastic force even in that situation; or, as if the cartilage itself possessed a higher vitality, and, therefore, the cytoblastema retained its productive power for a longer period, although penetrating quite as slowly as in the previous class.

Although the modifications in the form of the cells of this class vary but slightly from those of the preceding one, yet we see two striking changes in the cells and their cytoblastema, namely, the coalescence of the cell-walls and ossification. The thickening and transformation of the cell-walls were very distinct in the last class, for example, in feathers. Here a still more strongly-marked thickening of the cell-walls takes place in several cartilage cells. The external contours of the walls, however, gradually disappear in such instances, and a coalescence takes place to such an extent as to leave merely the cell-cavities perceptible, lying in an homogeneous substance. The blending of the cell-walls takes place either between the walls of neighboring cells, in instances where they are in immediate contact, or, with the intercellular substance, when the cells are surrounded by it. Further investigations are required in order to decide the question as to whether this blending be so complete that it cannot in any way be dissolved, the simple fact being, that the cell-walls are no longer discernible with the microscope. I shall not bring forward the splitting of the dental fibres as examples here, nor indeed make any reference to the teeth in this retrospect, their explanation being as yet too problematical. It has, however, been already mentioned as a doubtful point, whether a coalescence of the walls actually takes place in all cartilage-cells, for instance, in those of the higher animals.

Ossification appears to occur especially, perhaps exclusively, in those cartilages which have a greater quantity of intercellular substance. It consists, probably, in a chemical union between the calcareous salts and the firm portion of the cartilage substance. In the first commencement of the process, the cartilage frequently acquires a granulous appearance, which subsequently disappears, the entire substance meanwhile becoming gradually dark. At the same time, the cartilage-cells undergo a transformation into the osseous corpuscles, a process which must probably be explained as analogous to the formation of the stellated pigment-cells. There is reason to suppose that the osseous corpuscles and the canaliculi which issue from them, also become filled with earthy salts by the calcifying process.

The class of cells now under consideration, has yet another point of especial interest for us, since it is the first in which organized structures, that is, structures provided with vessels, occur. The accordance between the elementary cells of unorganized animal tissues and vegetable cells might be conceded, without granting a connexion between the organized tissues, (which are especially characteristic of animals,) and the structure of vegetables. A distinction had always been drawn between the growth of the organized and that of the unorganized structures; and much has already been said in a general way, about a vegetative growth of the non-vascular structures, the crystalline lens, for instance, though the analogy which existed between their elementary particles was not proved. Cartilage, then, is the first structure which teaches us that a tissue, which, at a later period at least, contains vessels, is composed of cells, perfectly according, in their development, with those of plants; and, therefore, that a similar formative principle is the basis both of the organized and unorganized tissues. We shall have further evidence of this presented to us in the following classes, which comprise the rest of the tissues—those, indeed, which are most perfectly organized, and the most important to the animal organism. In them we shall also find that the formation of cells is the general principle of development, and that their elementary particles are derived from cells, although, at the first glance, one would scarcely imagine that any connection could exist between them and cells.

